# Pressure Shift Freezing as Potential Alternative for Generation of Decellularized Scaffolds

**DOI:** 10.1155/2013/693793

**Published:** 2013-05-30

**Authors:** S. Eichhorn, D. Baier, D. Horst, U. Schreiber, H. Lahm, R. Lange, M. Krane

**Affiliations:** ^1^German Heart Center Munich, 80636 Munich, Germany; ^2^Institute for Food Biotechnology and Process Engineering, Technical University Berlin, 14195 Berlin, Germany; ^3^Institute of Pathology, LMU Munich, 80337 Munich, Germany

## Abstract

*Background*. Protocols using chemical reagents for scaffold decellularization can cause changes in the properties of the matrix, depending on the type of tissue and the chemical reagent. Technologies using physical techniques may be possible alternatives for the production grafts with potential superior matrix characteristics. *Material and Methods*. We tested four different technologies for scaffold decellularization. Group 1: high hydrostatic pressure (HHP), 1 GPa; Group 2: pressure shift freezing (PSF); Group 3: pulsed electric fields (PEF); Group 4: control group: detergent (SDS). The degree of decellularization was assessed by histological analysis and the measurement of residual DNA. *Results*. Tissue treated with PSF showed a decellularization with a penetration depth (PD) of 1.5 mm and residual DNA content of 24% ± 3%. HHD treatment caused a PD of 0.2 mm with a residual DNA content of 28% ± .4%. PD in PEF was 0.5 mm, and the residual DNA content was 49% ± 7%. In the SDS group, PD was found to be 5 mm, and the DNA content was determined at 5% ± 2%. *Conclusion*. PSF showed promising results as a possible technique for scaffold decellularization. The penetration depth of PSF has to be optimized, and the mechanical as well as the biological characteristics of decellularized grafts have to be evaluated.

## 1. Introduction

Currently, major drawbacks of prosthetic heart valves or artificial patch material, as well as aortic allografts, are lack of remodeling and growth potential and also degeneration and calcification [1, 2]. Tissue engineering is a promising technology to overcome the current limitations of existing prosthetic valves. Decellularized allogenic or xenogenic tissue is one of the preferred scaffold matrices for cardiovascular tissue engineering [3–5]. One major goal of ongoing research is implanting these matrices into the body having a curing effect. These decellularized matrices could be either seeded with autologous cells *ex vivo* before implantation, or the decellularized matrix will be implanted with subsequent cell seeding by circulating cells. There is no doubt that at the moment seeding at all implant sites and creating really functional tissue are still problems that have to be improved. The durability, cell seeding efficiency, and the graft viability reflecting the growth potential of tissue-engineered grafts mainly depend on the quality of the decellularized scaffolds. 

Many techniques for tissue decellularization have been reported [6–11]. The optimal decellularization protocol should result in a strongly reduced/absent antigenicity by removing the cellular components of the donor while preserving the extracellular matrix, and therefore the physiological biomechanical strength. Most treatments for decellularization are based on chemical agents like detergents [3, 6, 7, 9, 12–14]. Detergent treatment is easy to use but can have a long treatment time, residual toxicity, and a decreased biomechanical stability, dependent on the type of tissue, chemical agent, the concentration, and the exposition time [15]. In particular, regarding tissues with a very heterogenic matrix, high mechanical stability and elasticity, like the aorta, long treatment times, or high concentrations of chemical agent might be needed to achieve a sufficient decellularization, which can influence the biological, as well as, the mechanical matrix properties.

**Figure 1:**
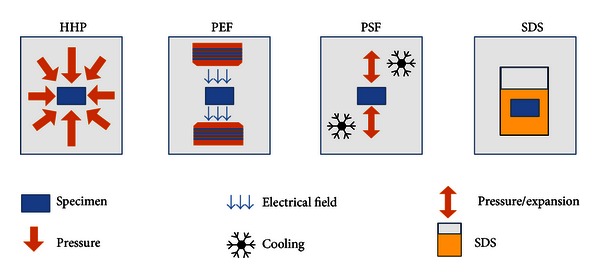
Different techniques used to treat the samples. Specimen and physical/chemical effects influencing the tissue structure are represented.

Treatment techniques using physical methods for tissue decellularization could be a conceivable alternative for scaffold decellularization with superior biological and mechanical properties and without residual toxicity compared to detergent-based decellularization. The aim of this study was to evaluate the techniques of high hydrostatic pressure, pulsed electric fields, and pressure shift freezing as potential alternative treatment options for an efficient aortic tissue decellularization. An overview of the different techniques used for decellularization is given in Figure 1.

## 2. Material and Methods

Fresh porcine aortic tissue was obtained from the local abattoir (MRT Ludwig Leidmann GmbH, Munich, Germany). The tissue was cleaned and stored immediately after harvesting at 4°C in phosphate buffered saline for transport to the laboratory for further processing. The aortic vessels were cut into pieces according to Fitzpatrick et al. [13] of 5 mm × 15 mm and were divided into 4 groups for decellularization treatment. Every group consisted of 5 samples. 

### 2.1. Treatment with Pulsed Electric Fields (PEF)

Pulsed electrical field technique is also known for decontamination of scaffolds or for temporary perforation of cell membranes [16]. The pulsed electric field unit used was constructed at the Technische Universität Berlin. Treatments were conducted in cuvettes with a sample volume of 800 *μ*L and two parallel aluminium electrodes with 4 mm distance for power transmission to the sample. Samples were treated in phosphate buffered saline to achieve a conductivity during treatment of approximately 15 mS/cm. Power supply of 11 kV resulted in voltages detected in the cuvettes of 9.1 kV and electric field strengths of 22.75 kV/cm. 130 pulses with a frequency of 2 Hz and a pulse width of 3 *μ*s were applied leading to total energy inputs of 128 kJ/kg. All treatments were performed at room temperature. 

### 2.2. Pressure Shift Freezing (PSF)

Pressure shift freezing is a technique that is primarily used in food processing industry for microbe inactivation [17]. High-pressure treatments with phase transition were performed in a high-pressure-low-temperature unit developed at the Technische Universität Berlin. The unit contains a vessel with a volume of 265 mL and a maximum pressure of 6600 MPa. The vessel is equipped with a double jacket connected to a thermobath which enables treatment temperatures from −50 to 100°C. Ethanol (80% v/v) was used as a cooling and pressure transmitting medium. Samples were treated in 0.9% NaCl solution in Cryovials (1.6 mL, Nunc Brand Products, Roskilde, DK). Treatments were performed at 200 MPa initial pressure and at −35°C, and samples were undercooled to −30°C after phase transition.

### 2.3. High Hydrostatic Pressure (HHP)

High hydrostatic pressure treatment was performed according to Funamoto et al. The vessel samples were pressurized using an isostatic pressure machine (Technische Universität Berlin, Germany) according to Funamoto et al. [18]. The specimens were placed in a Cryovial (Nunc Brand Products), and the thread was additionally sealed with parafilm (Pechiney Plastic Packaging, IL, USA). Afterwards, the samples were immersed in the transmission fluid of the pressure chamber and pressurized with a rate of 196 MPa/min at 30°C starting temperature until the pressure reached 980 MPa. The pressure was maintained for 10 min and then decreased at 196 MPa/min until atmospheric pressure was reached.

### 2.4. Detergent Treatment (SDS)

A solution containing 0,1% SDS (sodium dodecyl sulfate, (SDS Sigma Aldrich, Munich, Germany) together with RNase A (20 mg/mL, Sigma Aldrich, Munich, Germany) DNase (0,2 mg/mL, Sigma Aldrich), and 1% penicillin/streptomycin (PAA Laboratories, Marburg, Germany) in phosphate buffered saline was used for decellularization of the specimen [2]. The tissue was treated for 24 h at room temperature under constant agitation. After treatment, all samples of every treatment procedure were washed for 48 hours at 4°C in PBS containing 1% penicillin/streptomycin under continuous motion. This decellularization method was also used by Funamoto et al. as a reference treatment group.

### 2.5. Histology

For histological examination, longitudinal slices of the tissue were fixed in buffered formalin and embedded in paraffin. For the assessment of the general morphology, 4 sections (4 *μ*m each) were taken of each slice. The sections were stained with haematoxylin/eosine. Histological examination was performed using high-power light field microscopy. The main focus was the detection of residual cell nuclei and the orientation and integrity of the remaining collagen matrix.

### 2.6. Residual DNA Estimation

DNA was purified from 25 mg lyophilized tissue of each sample using the DNEasy Kit (Qiagen GmbH, Hilden, Germany) according to the manufacturer's guidelines. Five samples per treatment technique were taken. The DNA amounts and integrities were determined by calculation of the 260/280 nm ratios. Average DNA content and standard deviation were calculated in %, compared to the DNA content of a native tissue sample.

## 3. Results

### 3.1. Histological Examination

**Figure 2:**
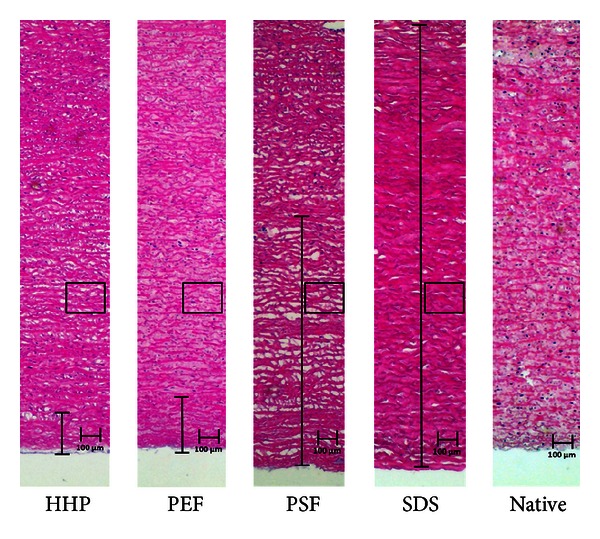
Illustration of the penetration depth (absence of cell nuclei) of the different treatment techniques compared to native tissue. In the rectangles, the areas that are shown in Figures 3 and 4 are marked.

In the control group treated with SDS, all cell nuclei were removed (Figure 2). The collagen fibers were oriented in parallel, and the extracellular matrix (ECM) remained homogeneous but appeared engorged and spongiform. 

**Figure 3:**
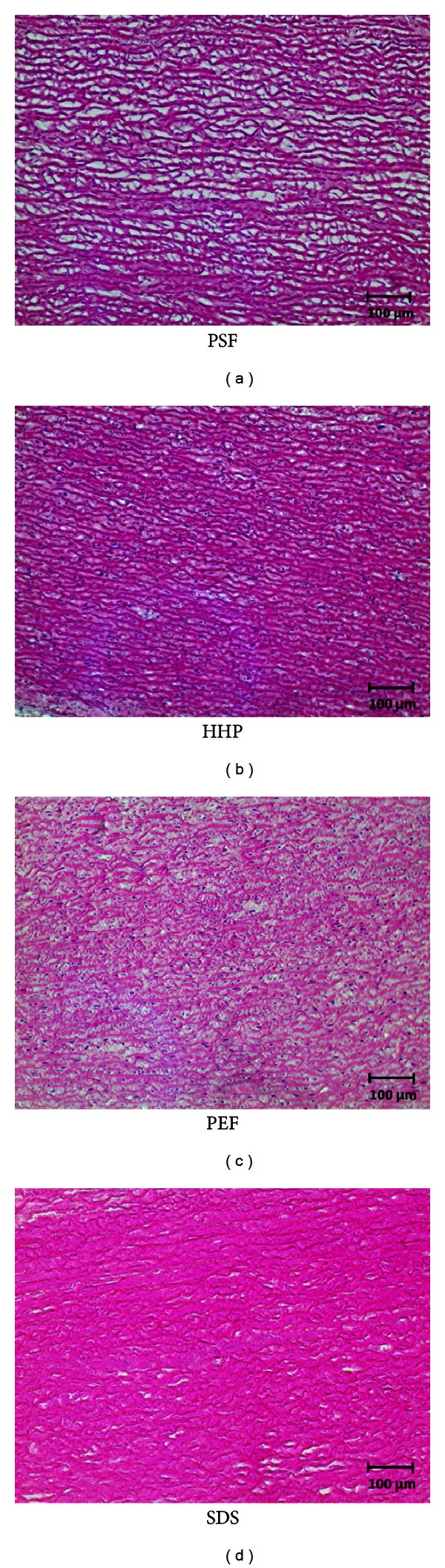
Images of the HE-stained sections (200x Magnification). In PSF-and-SDS treated tissue, all nuclei had successfully been removed. In PEF- and HHP-treated tissue, nuclei could still be detected.

**Figure 4:**
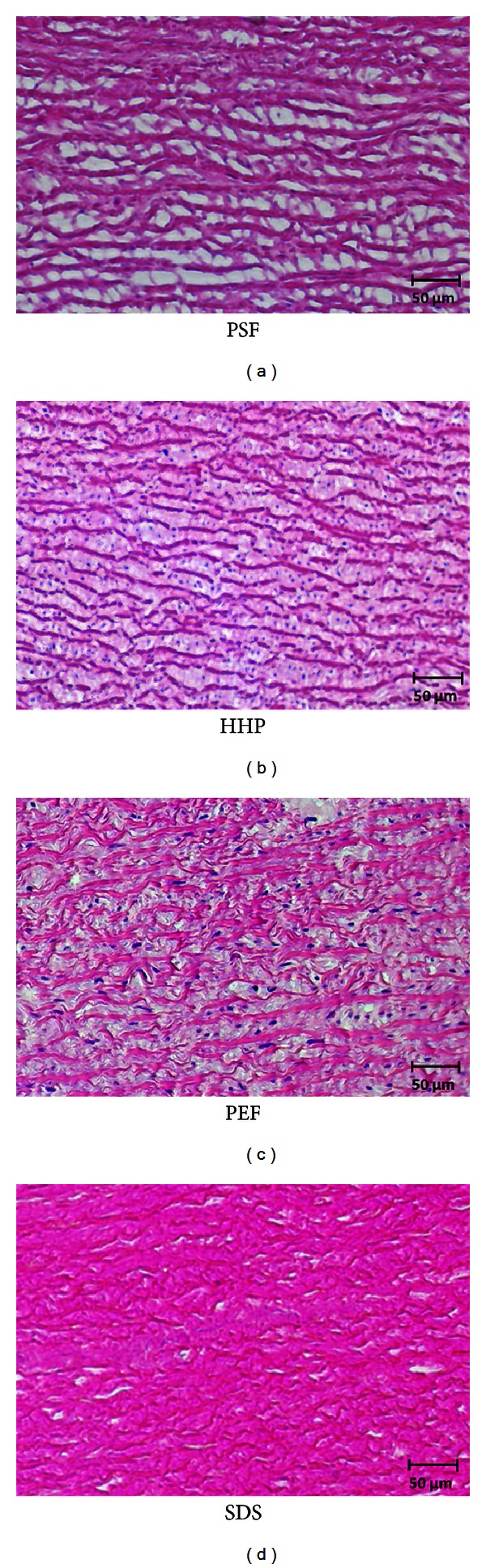
Images of the HE-stained sections (400x magnification). The expansion of the space between the collagen fiber filaments can be seen in the tissue treated with PSF.

HHP treatment did not yield a complete decellularization of the aortic tissue. During histological analysis, no cell nuclei could be detected within a penetration depth of 0.2 mm (Figure 2). After 0.3 mm, multiple cell nuclei could be detected in the samples. Furthermore, the collagen structure of the ECM was dissected in small fragments (Figures 3 and 4).

PSF leads to a complete decellularized scaffold with an average penetration depth of 1.5 mm (Figure 2). The ECM showed a more loosely but intact and parallel orientated structure of the collagen fibers. Detailed microscopic images of the histological sections are shown in Figures 3 and 4.

Treatment with PEF showed an average penetration depth of 0.5 mm (Figure 2). PEF treatment caused massive delamination of the whole extracellular matrix, and collagen fibers appeared dissected into multiple parts (Figures 3 and 4). 

### 3.2. Quantification of Residual DNA

**Figure 5:**
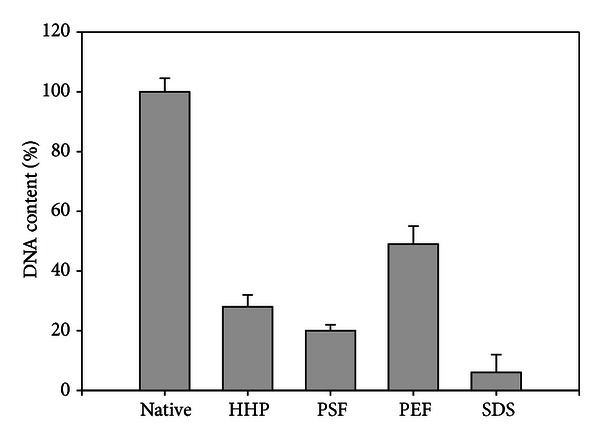
Average residual DNA content of the analyzed specimen treated with various techniques.

The residual DNA content was measured to further quantify the degree of decellularization achieved by the different physical technologies. It was quantified in relation to the native, untreated tissue samples. Compared to the native samples, treatment with SDS leads to a residual DNA content of 5% ± 2%. The treatment with HHP, PSF, and PEF reduced the residual DNA to 28% ± 4%, 24% ± 3%, and 49% ± 7%, respectively (Figure 5).

## 4. Discussion

At present, decellularization using detergents can be regarded as the gold standard to generate allogenic or xenogenic grafts for tissue engineering. Using SDS treatment, an almost complete decellularization could be achieved, but mechanical and structural as well as biological properties of the treated tissues are discussed to be inferior compared to native tissue [14, 15, 19]. Depending on concentration and exposition time, the influence of the treatment on the properties of the remaining matrix is variable. Very rigid tissues like the aorta need a higher concentration of chemical agents or a longer exposition time than “thin tissues” like cornea. Regarding the histological results, in our case, the concentration and the exposition time might influence the biological and the mechanical properties of the remaining matrix, which has to be investigated in further examinations. Different groups reported a complete decellularization of vessels and corneal tissue using HHP treatment [18, 20]. In our experiments, decellularization using HHP showed a limited penetration depth of 0.2 mm. Additionally, the collagen fibers of the remaining ECM that emerged were dissected by this treatment. This may be caused by the high rigidity of the pig's aortic tissue. Additionally, the other groups used thinner tissue slices, leading to a better penetration depth than we did. Another difference in the protocol that has to be mentioned is the use of cryotubes instead of plastic bags. In our opinion, due to the physical properties of the high hydrostatic pressure, this will cause no differences in the effects of the treatment process. The use of cryotubes in high hydrostatic pressure experiments has also been described by other authors [21, 22]. Moreover, decellularization treatment using PEF was only partially sufficient based on the histological examination. The remaining collagen fibers were also dissected, and the penetration depth was limited to 0.5 mm. In contrast, PSF seemed to be an interesting technique to generate decellularized scaffolds. Almost no nuclei could be detected by microscopic examination with a penetration depth of 1.5 mm. The increased space between the collagen fibers is probably caused by the crystallization process, which occurs during freezing intervals. The four- to fivefold higher residual DNA content compared to detergent treatment with SDS is most likely caused by the nondecellularized tissue area, which could not be reached by PSF treatment. The main focus for further studies has to be an improvement of the process parameters for PSF treatment to increase the penetration depth. One possible way could be repeating the treatment for several cycles. Once the reduction of residual DNA is comparable to the SDS technique, it has to be investigated, whether the morphological, immunological, and mechanical properties are comparable or superior to tissue that has been decellularized with detergents.

## 5. Conclusion

PSF treatment seems to be an interesting technique for decellularization of tissue. Further studies have to investigate the mechanical and immunological characteristics of decellularized scaffolds following PSF treatment. Additionally, the influence of the more loosely organized ECM regarding the ingrowth behavior of seeded cells has to be evaluated. Although PSF seems to be an alternative for tissue decellularization up to a penetration depth of 1.5 mm, future work has to focus on the extension of the penetration depth for decellularization of tissues with larger diameters before mechanical and immunological examinations can be performed.
